# Annexin-A1 Regulates MicroRNA-26b* and MicroRNA-562 to Directly Target NF-κB and Angiogenesis in Breast Cancer Cells

**DOI:** 10.1371/journal.pone.0114507

**Published:** 2014-12-23

**Authors:** Durkeshwari Anbalagan, Gracemary Yap, Yi Yuan, Vijay K. Pandey, Wai Hoe Lau, Suruchi Arora, Pradeep Bist, Justin S. B. Wong, Gautam Sethi, Peter M. Nissom, Peter E. Lobie, Lina H. K. Lim

**Affiliations:** 1 Department of Physiology, Yong Loo Lin School of Medicine, National University of Singapore, Centre for Life Sciences, 28 Medical Drive, Singapore, 117456, Singapore; 2 Cancer Science Institute, 14 Medical Drive, #12-01, Centre for Translational Medicine, MD6 Singapore, 117599, Singapore; 3 Department of Microbiology, Yong Loo Lin School of Medicine, National University of Singapore, Centre for Life Sciences, 28 Medical Drive, Singapore, 117456, Singapore; 4 Department of Pharmacology, Yong Loo Lin School of Medicine, National University of Singapore, MD11, 10 Medical Drive, Singapore, 117597, Singapore; 5 Astar-Bioprocessing Technology Institute, 20 Biopolis Way, 138668, Singapore, Singapore; 6 NUS Immunology Program, Life Sciences Institute, Centre for Life Sciences, 28 Medical Drive, Singapore, 117456, Singapore; Sun Yat-sen University Medical School, China

## Abstract

Annexin 1 (ANXA1) is an endogenous anti-inflammatory protein implicated in cancer. ANXA1 was previously shown to be regulated by hsa-miR-196a. However, whether ANXA1 itself regulates microRNA (miR) expression is unknown. Therefore, we investigated the regulation of miR by ANXA1 in MCF7 breast cancer cells. MCF7-EV (Empty vector) and MCF7-V5 (ANXA1-V5 expressing cells) were subjected to a miR microarray. Microarray analysis revealed a number of miRNAs which were dysregulated in MCF7-V5 cells. 2 novel miRNAs (miR562 and miR26b*) were validated, cloned and functionally characterized. As ANXA1 constitutively activates NF-κB activity to modulate breast cancer metastasis, we found that miR26b* and miR562 directly targeted the canonical NF-κB pathway by targeting the 3′ UTR and inhibiting expression of Rel A (p65) and NF-κB1 (p105) respectively. MiR562 inhibited wound healing, which was reversed when ANXA1 was overexpressed. Overexpression of either miR562 or miR26b* in MCF-7 cells enhanced endothelial tube formation when cocultured with human umbilical cord endothelial cells while conversely, treatment of MCF7 cells with either anti-miR562 or anti-miR26b* inhibited endothelial tube formation after co-culture. Further analysis of miR562 revealed that miR562-transfected cell conditioned media enhances endothelial cell tube formation, indicating that miR562 increased angiogenic secreted factors from MCF-7 breast tumor cells. TNFα was increased upon overexpression of miR562, which was reversed when ANXA1 was co-transfected In conclusion, this data suggests that ANXA1-regulated miR26b* and miR562 may play a role in wound healing and tumor-induced endothelial cell tube formation by targeting NF-κB expression and point towards a potential therapeutic target for breast cancer.

## Introduction

NF-κB consists of an agglomeration of closely-related protein dimers and is a well-characterised transcription factor. The signalling paradigm of NF-κB has been broadly divided into classical and non-classical pathways. The canonical pathway plays important roles in innate immunity, inflammation and cell survival [1], [2] and is triggered by many many stimuli such as microbial and viral infections as well as proinflammatory cytokines. NF-κB has been reported to be constitutively activated in cancer [3]. NF-κB has been found to be involved in cancers of epithelial origin such as breast cancer. Many studies have reported elevated or constitutively active NF-κB DNA-binding activity in mammary carcinoma and primary breast cancer cells of human and rodent origin [4], [5], [6]. This indicates that constitutive NF-κB activation might be one of the early events in breast cancer progression. The caveat in systemic inhibition of NF-κB may affect global innate immune responses. Therefore, though NF-κB is an attractive therapeutic option, long-term inhibition is not feasible.

In that respect, Annexin A1 (ANXA1) is an anti-inflammatory protein implicated in affecting many cellular processes. We have previously shown that ANXA1 expression correlated with NF-κB activity. Further studies revealed that ANXA1 can bind to and interact with IKKγ (NEMO) but not IKKα or IKKβ and can recruit RIP1 to the IKK complex, indicating that ANXA1 is crucial for constitutive activation of NF-κB in breast cancer to promote metastasis [7]. The expression of ANXA1 has been profiled in many different cancer subtypes and showed considerable success as a possible prognostic and diagnostic marker in some cancer such as hairy cell leukemia and cholangiocarcinoma [8], [9]. The expression of ANXA1 was increased in certain cancers such as pancreatic cancer, and gastrointestinal cancer [10], [11] and decreased in others such as esophageal and prostate cancer [12], [13], [14]. Though expression of ANXA1 has been neatly correlated to tumour classification in some cancer subtype, reports on breast cancer have been conflicting and there is no consensus on expression of ANXA1 in breast cancer [15], [16], [17]. This may be due to the high degree of heterogeneity observed in breast cancer and the different types of breast cancer, ie basal or ductal carcinomas [18].

MicroRNAs (miRs) are a group of non-coding RNAs which have been shown to regulate many genes involved in cellular processes such as proliferation, differentiation and apoptosis [19]. Under the classical model, miRs recognise their target gene transcripts through a seed sequence of 2–8 nucleotides long and bind to their target gene transcript at the 3′ UTR of gene transcripts [20]. This binding interaction results in either mRNA degradation of the gene transcripts or inhibition of translation. As miRs have been reported to regulate many genes, their involvement in tumorigenesis is not surprising. Thus, miR profiling has often been reported in tumour classification, diagnostics and therapeutics [21], [22], [23]. With respect to ANXA1, it is a target of HSA-miR196a [24] and the expression of hsa-miR-196a is inversely correlated with ANXA1 expression in esophageal, breast and endometrial cancer cell lines. MiR-196a specifically targeted ANXA1 and promoted cell proliferation and anchorage-dependent growth and suppressed apoptosis.

As ANXA1 can regulate transcription factors and downstream gene activation, we speculated that ANXA1 could also regulate microRNA expression. Therefore, in this study, we investigated if ANXA1 could regulate miRNAs, and if these miRs could target certain factors of NF-κB pathway and modulate NF-κB activity and downstream function in cancer cells.

## Materials and Methods

### Cell Culture

Human breast cancer cell line MCF7 were obtained from ATCC. ANXA1-V5 was stably transfected into MCF7 cells as described previously [7]. The cells were grown as monolayers in Dulbecco's Modified Eagle's Medium (DMEM) containing with 10% v:v heat-inactivated fetal bovine serum (FBS, Biowest), 2 mM L-glutamine (GIBCO) and 100 U/ml penicillin and 100 µg/ml streptomycin (Hyclone) at 37°C in a humid atmosphere containing 5% CO_2_. For cells overexpressing ANXA1, a pcDNA3.1-V5 plasmid expressing human ANXA1 (kindly provided by Fulvio D′Aquisto, William Harvey Research Institute, London) was stably transfected into MCF-7 cells using G418 selection. Stable clones were selected and grown. A control set of cells was transfected with an empty pcDNA3.1 vector. Human umbilical cord endothelial cells (Huvec) cells obtained from Lonza Clonetics Endothelial Cell Systems were cultured in Endothelial Growth Medium-2 (EGM-2) supplemented with EGM-2 Bulletkit containing 2% FBS, Hydrocortisone, hFGF-B, VEGF, R3-IGF-1, Ascorbic Acid, Heparin, FBS, hEGF, GA-1000. Cells were grown in a humid atmosphere containing 5% CO_2_ at 37°C.

### Isolation of miR

Enriched miR was isolated from cell pellets using mirPremier miR isolation kit (Sigma) according to the manufacturer's protocol. The enriched miR was quantified using a NanoDrop spectrophotometer (NanoDrop Technology) where 1 A260 unit is 33 µg/ml of small RNA. The integrity of the miR was determined using an Agilent Bioanaylzer (Agilent Technology).

### MiR Microarray profiling and analysis

MiRNA microarray analysis was performed using the Affymetrix Solution GeneChip MicroRNA Version 1.0 array (comprising 46228 probes with 7815 probes sets) according to manufacturer's (Affymetrix) instructions. Briefly, a starting material of 1 µg of small RNA was used for poly A tailing on the 3′ end of the small RNA followed by ligation of the biotinylated signal molecule to the small RNA samples, according to Affymetrix Flashtag RNA labeling kit (Affymetrix, p/N FT10AFYB). Hybridzation procedure was performed as described in the manufacturer's protocol. The chips were scanned using Affymetrix Genechip Scanner 3000 and the data was extracted using Genechip Operating Software (GCOS).

The data discussed in this publication have been deposited in NCBI's Gene Expression Omnibus and are accessible through GEO Series accession number GSE54439.

### RNA Isolation and qPCR

Total cellular RNA was isolated with TRIzol (Invitrogen) according to manufacturer's instructions. Poly A tailing was only performed in samples where qPCR for quantification of miR levels was done using the Poly A tailing kit (New England Biolabs) according to manufacturer's instructions. 1 µg of total RNA was transcribed using Promega (for non-miR qPCR) or Tiangen (for miR qPCR) Reverse Tanscription kits according to manufacturer's instructions. qPCR was performed using GoTaq qpCR master mix (Promega). The following PCR protocol was used: 50°C for 2 min, 95°C for 10 min followed by 40 cycles of 95°C for 15 s, 60°C for 1 min, 50°C for 15s, 60°C for 1 min, 95°C for 30s, 60°C for 15s. Melting curve analysis was performed after that at 95°C for 1 minute and 55°C for 1 minute. The ABI 7500 real-time PCR system (Applied Biosystems) was used for analysis. Glyceraldehyde-3-phosphate dehydrogenase (GAPDH) was used as the endogenous control for non-miR qPCR reactions and hsa-miR-9* was used as the endogenous control and a universal reverse primer was used for miR qPCR. The sequence of the universal reverse primer was: 5′-CGAATTCTAGAGCTGGAGGCAGGCGACATGGCTGGCTAGTTAAGCTTGGTACCGA-3′. The primer sequence for hsa-miR26b* was 5′ CCTGTTCTCCATTACTTGGCTC-3′ and hsa-miR562 was 5′–AAAGTAGCTGTACCATTTGC-3′. The primer pairs used for non-miR qPCR are shown in Table 1, and all have equal efficiency. Triplicates were performed for each of the genes. No non-specific amplification or primer-dimers were observed. Relative expression was computed by ΔΔCt approximation method.

**Table 1:** Primer sequences used for quantitative PCR.

ANGPT1	FP: CTCGCTGCCATTCTGACTCAC
	RP: GACAGTTGCCATCGTGTTCTG
ANGPT2	FP: TGGGATTTGGTAACCCTTCA
	RP: GTAAGCCTCATTCCCTTCCC
NFKB1	FP: TGCCAACAGATGGCCCATAC
	RP: TGTTCTTTTCACTAGAGGCACCA
STAT5A	FP: TTCTTGTTGCGCTTTAGTGACT
	RP: TGGTGAATGGTTTCAGGTTCC
SERPINE1	FP: CACAAATCAGACGGCAGCACT
	RP: CATCGGGCGTGGTGAACTC
PLAU	FP: CACGCAAGGGGAGATGAA
	RP: ACAGCATTTTGGTGGTGACTT
MMP1	FP: AGCTAGCTCAGGATGACATTGATG
	RP: GCCGATGGGCTGGACAG
MMP9	FP: TGGGGGGCAACTCGGC
	RP: GGAATGATCTAAGCCCAG
p53	FP: TGCAGCTGTGGGTTGATTCC
	RP: AAACACGCACCTCAAAGCTGTTC
S100A4	FP: GATGAGCAACTTGGACAGCAA
	RP: CTGGGCTGCTTATCTGGGAAG
FOS	FP: TGCCTCTCCTCAATGACCCTGA
	RP: ATAGGTCCATGTCTGGCACGGA
ERBB2	FP: ACTGGCCCTCATCCACCATA
	RP: GGTTGGCAGTGTGGAGCAG
Rel A	FP: TTGAGGTGTATTTCA CGGGACGACC
	RP: GCACATCAGCTTGCG AAAAGG
TGFB1	FP: GCCCTGGACACCAACTATTG
	RP: CGTGTCCAGGCTCCAAATG
TNF	FP: CCAGGCAGTCAGATCATCTTCTC
	RP: AGCTGGTTATCTCTCAGCTCCAC
IL-8	FP: GAATGGGTTTGCTAGAATGTGATA
	RP: CAGACTAGGGTTGCCAGATTTAAC

### Cloning of primary miR transcripts from genomic DNA

Human blood peripheral monocytes were obtained from human volunteers under written informed consent approved by the Institutional Board of the National University of Singapore. Cells were pelleted down and for a fully confluent 10 cm^2^ dish, 1 ml of the red blood cell lysis buffer (0.01 M Tris-HCl pH 7.6, 320 mM sucrose, 5 mM MgC1_2_, 1% Triton ×100) was added to the cell pellet. The pellet was resuspended well and centrifuged at 2,700xg for 2 minutes. Supernatant was discarded and 400 µl of nucleic acid lysis buffer (0.01 M Tris-HC1, 11.4 mM sodium citrate, 1 mM EDTA, 1% SDS) was added to resuspend the pellet. To the resuspension, 800 µl of ice-cold absolute ethanol was then added. Mixture was shaken gently and then vortexed briefly. Samples were centrifuged at 12000xg for 1 minute. Supernatant was discarded and tubes were allowed to air-dry completely. DNA was resuspended in TE buffer and stored at −20°C.

The sequences of the primary transcripts of the miRs were extracted from NCBI. These sequences were typically about 100 bases long. Flanking sequences of about 200 bases were added to the beginning and end of primary transcript sequence. Primers were then designed to amplify this entire region of 500 bases. Primary miR transcripts were amplified from genomic DNA extracted from control cells. The amplified insert was subjected to BamHI-HindIII (Promega) restriction enzyme (RE) digestion. This was followed by ligation and transformation of DNA into E coli DH5α competent cells. Successful clones were sent for sequencing and sequence homology was checked with the primary transcript sequence using nucleotide blast (http://blast.ncbi.nlm.nih.gov/Blast.cgi?PROGRAM=blastn&PAGE_TYPE=BlastSearch&LINK_LOC=blasthome). The quality of DNA (A260/A280 ratio) and quantity of DNA was determined using a nanodrop spectrophotometer (Biofrontier Technology). Only DNA samples with A260/A280 ratio of values equal to or greater than 1.8 were used and DNA was stored at −20°C.

### Cloning of 3′ UTR of putative targets of miRs

The protocol of cloning the 3′ UTR of putative miR targets was similar to the protocol of cloning miRs from genomic DNA with slight differences as the starting material was cDNA and no additional flanking sequences were added to the sequence. The sequence of 3′ UTR of RELA (NM_001145138) and NF-κB1 (NM_003998) were retrieved from NCBI (http://www.ncbi.nlm.nih.gov/nuccore). Primers were designed based on this sequence. QuikChange Site-directed Mutagenesis kit (Stratagene) was used to mutate 3 out of 5 binding nucleotides of seed sequence of Rel A and NF-κB1. Manufacturer's protocol was used.

### Luciferase Assay

Cells were transfected when they reached 50% confluency. The cells were transfected with NF-κB luciferase reporter plasmid (Stragene) and Renilla plasmid in serum-free medium with and without miRs. Cells were incubated for 24 hours after which cells were treated with phorbol 12-myristate 13-acetate (PMA) (20 ng/ml) for 6 hours. Luciferase activity was determined using Dual-Luciferase Reporter Assay System (Promega). The results were expression as relative NF-kB promoter luciferase activity compared to controls after normalising for Renilla activity and protein concentration. Luminescence was measured using a spectrophotometer (Perkin Elmer VICTOR3 V Multilabel Counter Model 1420).

### Determination of cell viability

Cells were transfected with varying doses of miRs as per manufacturer's instructions. 48 hours post transfection, cells were lifted with 1× trypsin for 5 minutes at 37°C. Cells were counted and 5000 cells were seeded per well (96 well format). Cells were allowed to grow for 5 days before cell viability was assessed by CellTiter-Glo Luminescent Cell Viability Assay (Promega). Alternatively, cell viability for population doubling studies was determined using crystal violet staining. Cell culture medium was aspirated and cells were washed once with 1× PBS (containing 137 mM NaCl, 2.68 mM KCl, 10 mM NaHPO4 and 2 mM KH2PO4). Crystal violet solution (0.25 g crystal violet powder in 20% methanol and 80% PBS) was added to each well. Crystal violet solution was gently removed and cells were washed. 1% SDS was added to each well and absorbance was read using a spectrophotometer at 570 nm. For PI staining, cells were treated with a buffer containing RNase A and propidium iodide (PI) after ethanol fixation. The treatment buffer contained 100 µg/ml RNase A (from stock solution), 0.1% Triton-X in PBS (1% solution was prepared) and 20 µl of 10 mg/ml PI solution in PBS for 30 ml treatment buffer.

### Wound healing assay

Cells were transfected with miRs according to manufacturer's (Qiagen) protocol. 24 hours after transfection, a ‘scratch’ was made in the cell monolayer to create a wound. Wound healing was observed at 0, 6, and 24 hours under light microscope at 10× magnification and photos were taken. Wound healing was analysed using Image J software (National Institutes of Health).

### Tube formation assay

Human umbilical cord endothelial cells (Huvec), were seeded onto Matrigel Matrix Growth Factor Reduced (BD Biosciences) in serum-free medium. MCF7 cells were transfected according to manufacturer's (Qiagen) protocol (either miR overexpression or knockdown). Cells were trypsinised and seeded into the cell culture insert (0.4 µm pores) in DMEM supplemented with 10% FBS for 24 hours. Matrigel-cells layer was washed and fixed with 4% paraformaldehyde. Total tubule length and number were analysed by Image J software (National Institutes of Health).

### Statistical data analysis

Unpaired sets of data were compared using unpaired Student's *t*-test (two-tailed). In all cases, *P<0.05* was accepted as statistically significant.

## Results

### ANXA1 regulates miR expression profiles in breast cancer

**Figure 1:**
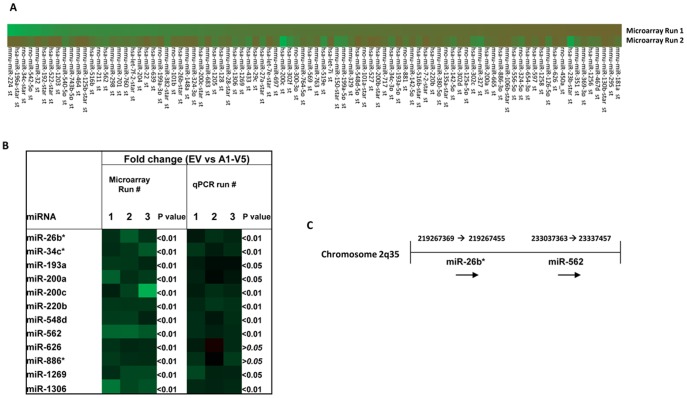
Microarray and qPCR validation of microRNA dysregulation in MCF-7 cells overexpressing ANXA1. (A) Heat map showing pattern of dysregulation observed in miRs during miR microarray analysis when ANXA1 was over-expressed in MCF7 cells. Green bars represent down-regulation of miR expression and red bars represent up-regulation of miR expression. The intensity corresponds to the degree of dysregulation of expression compared to MCF7-EV cells. (B) Correlations between 3 microarray runs and 3 qPCR validations of 12 miRs chosen. p values shown are vs MCF7-EV control cells. (C) Chromosome location of miR26b* and miR562. (E) miR26b* and (F) miR562 expression in breast cancer cell lines. * p<0.05 ** p<0.01 vs MCF10A cells

In order to investigate if and which miRs are dys-regulated when ANXA1 is over-expressed, we analyzed a stable, functional over-expressing ANXA1 model in MCF7- breast cancer cells, (A1–V5, stably ANXA1 over-expressing cells), previously published in [7] which were subjected to miR microarray analysis alongside control MCF7-EV (pcDNA3.1 empty vector) cells. 159 miRs were found to be differentially expressed in A1–V5 MCF7 cells as compared to MCF-7-EV cells (Fig. 1A). 12 miRs which displayed consistent differential expression patterns as compared to the MCF7-EV cells over three runs in the microarray analysis, were chosen and validated by real-time PCR (Fig. 1B). Subsequently, the precursor forms of hsa-miR26b*(miR26b*) and hsa-miR562 (miR562) were selected and cloned from human blood peripheral monocytes. miR26b* is located on chromosome 2 at co-ordinates 219267369–219267455 and miR562 is located on the same chromosome 2 at co-ordinates 233037363–233037457 (Fig. 1C). Interestingly, miR26b* is the passenger strand (3p) produced together with hsa-miR26b.

**Figure 2:**
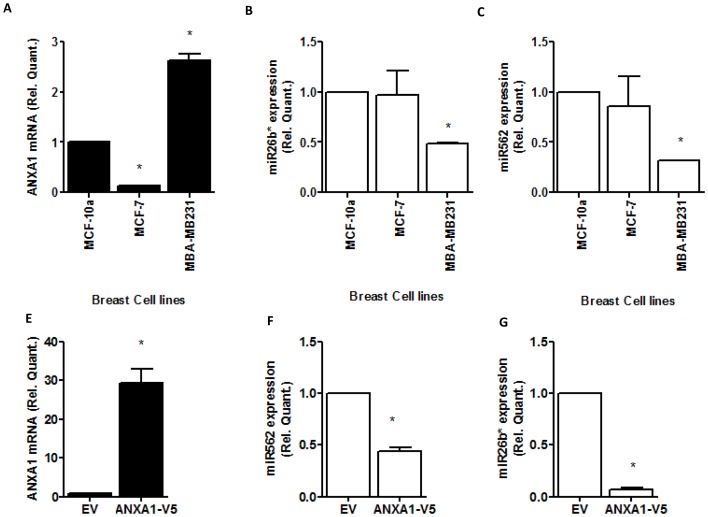
Expression of miR26b* and miR562 in breast cancer cells. (A–C) RNA from MCF10A breast epithelial cells, MCF7 and MDA-MB231 breast cancer cells were isolated and expression levels of ANXA1, miR26b* and miR562 were measured. (D–E) MCF7 cells were stably transfected with a ANXA1 overexpression vector and levels of ANXA1, miR26b* and miR562 were assessed. * p<0.05 ** p<0.01 vs MCF-10A cells or EV. Values are represented as fold change vs MCF-10A cells or EV.

We next confirmed that ANXA1 could inhibit the expression of these 2 microRNAs in MCF7 cells. Fig. 2A–C show that miR26b* and miR562 expression are reduced when ANXA1 is high. In addition, we assessed the endogenous levels of these miRs in breast cancer cell lines MCF7, which have been shown to express low ANXA1 [7] and MDA-MB-231, which express high levels of ANXA1 [7] together with a breast epithelial cell line, MCF10a. Lower levels of miR-26b* (Fig. 2E) and miR562 (Fig. 2F) were expressed in MDA-MB231 with no significant change in MCF-7. These data confirm that high levels of ANXA1 correlate with low levels of miR-26b* and miR562.

### miR26b* and miR562 regulate NFκB activity in breast cancer cells

**Figure 3:**
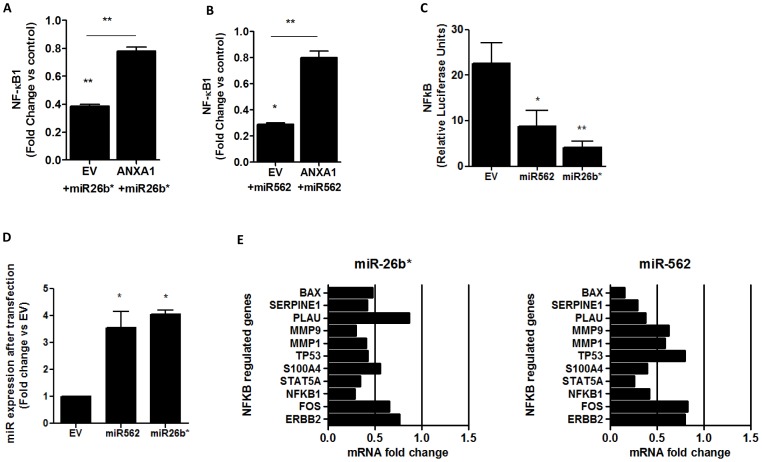
miR26b* and miR562 overexpression modulates NF-κB activity. (A,B) qPCR expression of NFKB1 after miR26b* and miR562 overexpression in MCF7 and MCF7-V5 cells (overexpressing ANXA1). (C) MCF7 cells were transfected with empty vector (p-sil), miR26b* or miR562 together with a NF-κB luciferase promoter and NF-κB luciferase activity was measured. * p<0.05 ** p<0.01 vs EV. (D) miR26b* and miR562 expression after transfection with plasmid (E) qPCR analysis of NFKB regulated genes after transfection with miR26b* or miR562. Values are represented as fold change vs EV.

As we have previously shown that ANXA1 can regulate NF-κB activity [7], we next elucidated if miR26b* or miR562 could be regulating NF-κB. NF-κB1 was inhibited after transfecting MCF-7 cells with miR26b* or miR562, and this inhibition was reversed in MCF7-V5 ANXA1 stably transfected cells (Fig. 3A,B). To confirm that NF-κB was indeed modulated by the 2 miRs studied, HEK 293T cells transiently expressing either miR26b* or miR562 were transfected with a construct harboring a NF-κB-binding site upstream of a luciferase reporter and stimulated with PMA. NF-κB-promoter activity was quantified by measuring luciferase activity. A decrease in fold-induction of NF-κB-promoter activity after PMA treatment was observed in cells over-expressing either miR26b* or miR562, indicating that miR26b* and miR562 was able to down-regulate NF-κB activity (Fig. 3C), while miRs were overexpressed (Fig. 3D). We next determined if NF-κB-dependent genes such as MMP1 or MMP9 were also downregulated in MCF-7 cells transfected with miR26b* or miR562 (Fig. 3D,E). qPCR analysis demonstrated that 7/11 NF-κB-dependent genes were downregulated to less than 0.5-fold after transfecting with miR26b*, and 6/11 NF-κB-dependent genes were downregulated to less than 0.5-fold after transfecting with miR562, confirming the inhibition of NF-κB activity.

MicroCOSM, a bioinformatics prediction tool, was used to predict putative targets of miR26b* and miR562 which were related to the NF-κB pathway. miR26b* was predicted to target Rel A, whose protein product is p65, at the 3′ UTR end of the gene transcript. The p65 subunit is one of the five NF-κB subunits, which dimerises with the p50 subunit under the classical NF-κB pathway and this dimer translocates to the nucleus to initiate transcription. With respect to miR562, NF-κB1 and NF-κB activating protein were two NF-κB related genes predicted as targets. NF-κB1 produces p105 protein which is the precursor of the p50 subunit which dimerises with the p65 subunit in the classical pathway.

### miR26b* and miR562 targets REL-A and NFκB1, respectively

**Figure 4:**
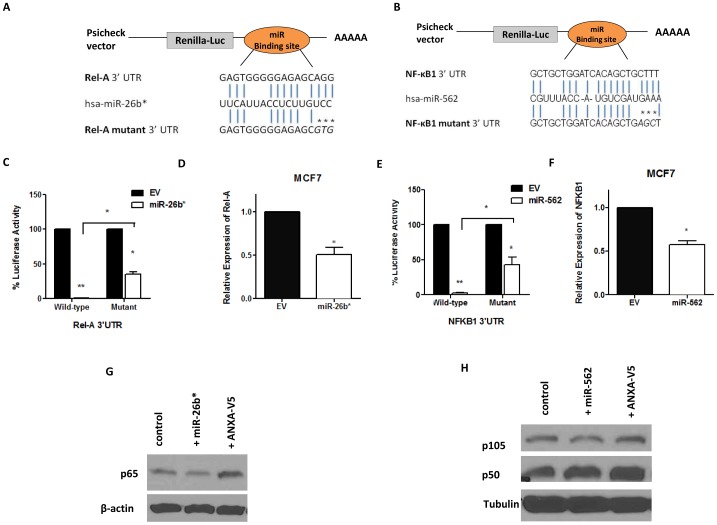
3′UTR cloning and target analysis of miR26b*/RelA and miR562/NF-κB1. WT and mutant (A) RELA and (B) NF-κB1 3′UTR were subcloned into psiCHECK-2 luciferase vector. The predicted miR binding sites within the respective 3′UTRs is shown. The mutated binding site is represented by asterisks (*). (C,E) 293T cells were transfected with the RELA or NF-κB1 3′UTR plasmid or the mutant RELA or NF-κB1 3′UTR plasmid together with their respective miRs. (D, F) PCR analysis of RELA or NF-κB1 expression after transfection with their respective miRs. (G, H) Western blot analysis of NF-κB protein products p65 and p105/50 after transfection with their respective miRs. A positive control of MCF7-V5 cells overexpressing ANXA1 is shown in lane 3.

To validate the bioinformatics prediction of putative targets, direct binding of miR to the 3′UTR of target gene transcript followed by analysis of RNA and protein expression of the putative target was performed. Hence, the 3′ UTR of Rel A and NF-κB1 were cloned into the psiCHECK-2 vector so that direct binding between miR and target gene transcript could be assessed. The seed sequences of both has-miR26b* and has-miR562 have 6 nucleotides, out of which 5 are binding nucleotides (Fig. 4A,B). Site-specific mutagenesis was performed to mutate the last 3 binding nucleotides of seed sequences of both miRs. In the 3′ UTR analysis of Rel A, luciferase activity was completely inhibited when miR26b* was co-transfected with the 3′ UTR of Rel A in HEK 293T cells, indicating complete binding of miR26b* to the 3′UTR of Rel A (Fig. 4C). A partial rescue (64.4±5.9% rescue) of luciferase activity was observed when miR26b* was co-transfected with the mutated Rel A 3′ UTR plasmid, suggesting that more nucleotides in the seed sequence may need to be mutated. The same observation was recorded in 3′ UTR analysis of NF-κB1 when miR562 was transfected into HEK 293T cells (56.7±10.7% rescue, Fig. 4E). These observations demonstrate that has-miR26b* and has-miR562 target Rel A and NF-κB1 at the 3′ UTR respectively.

To determine if the miRs can result in a reduction in transcription and translation, qPCR analysis and western blotting was performed after transfection of the miRs. RNA levels of Rel A decreased in MCF7 cells when miR26b* was transiently over-expressed in these cells (Fig. 4D). This was followed by decrease in protein levels of total p65 levels (Fig. 4F) which was accompanied by an increase in p65 protein levels in ANXA1-overexpressing MCF7 cells. Similarly, RNA levels of NF-κB1 were reduced in MCF7 cells transiently over-expressing miR562 (Fig. 4E). p105 protein levels was  =  reduced when miR562 was transfected into MCF-7 cells, with an increase when ANXA1 was overexpressed. This data confirm that miR26b* and miR562 directly target and down-regulate RNA and protein levels of Rel A/p65 and NF-κB1/p105, respectively.

We next investigated the functions of these 2 miRs on breast cancer proliferation, wound healing and tumor-induced endothelial tube formation. MCF7 cells transiently expressing either miR were quantified using crystal violet staining every day for 5 days. S1 Fig. illustrates that no difference in growth rates were observed when either miR26b* or miR562 were transfected under the conditions studied (S1 Fig.). To confirm this, cell cycle analysis was performed and once again, no difference in cell cycle progression was observed for both miRNAs, indicating that both miR26* and miR562 do not affect proliferation in MCF7 cells.

### Effects of miR26b* and miR562 on wound healing

**Figure 5:**
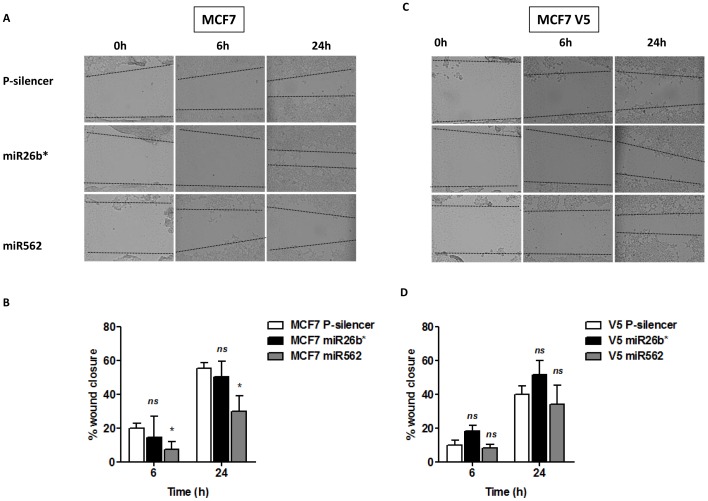
miR562 overexpression in MCF7 cells inhibits wound healing. (A) MCF7 cells were transfected with empty vector (EV), miR26b* or miR562 and wound healing was observed at 0 h, 6 h and 24 h. Representative pictures are presented and (B) % wound closure analyzed. (C,D) Similar experiments were performed with MCF7-V5 cells stably overexpressing ANXA1. *ns* not significant, * p<0.05 vs MCF7 P-silencer cells.

The effect of the miRs on migration of MCF7 cells was assessed by a wound healing assay where a scratch was made in the cell layer and wound closure was monitored over the course of 24 hours. A gradual increase in wound closure was recorded in both control and miR-transfected MCF7 cells (Fig. 5A). No significant difference was noted in wound closure between control and miR26b* cells, indicating that miR26b* did not affect migration in MCF7 cells. However, a significant inhibition of wound closure was observed after miR562 transfection, 6 h and 24 h after the wound was made (Fig. 5A,B). Interestingly, transfection of miR562 into ANXA1 overexpressing MCF7 cells reversed this inhibition in migration (Fig. 5C,D). This indicates again that ANXA1 down-regulates miR562, which may be involved in inhibition of wound healing in MCF7 breast cancer cells.

### miR26b* and miR562 enhance tumor cell induced endothelial cell tube formation

**Figure 6:**
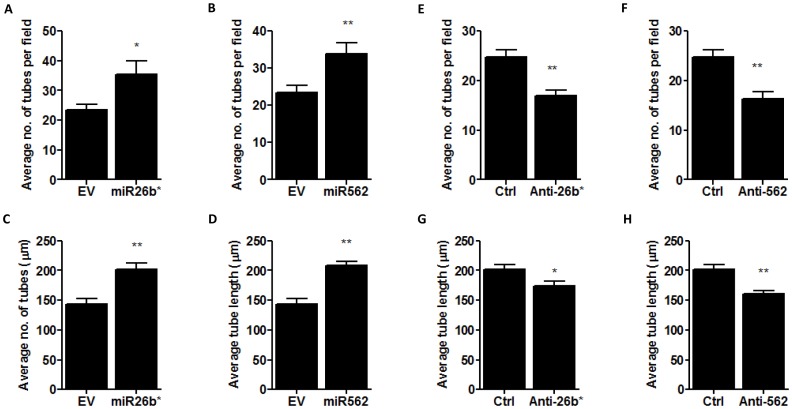
miR26b* and miR562 overexpression in MCF7 cells enhances endothelial cell angiogenesis while silencing miR26b* and miR562 inhibits angiogenesis. MCF7 cells were transfected with empty vector (EV), miR26b* or miR562 and a co-culture using transwells was performed with HUVEC in matrigel. (A,C) Average number of tubes formed per field of view and (B,D) average tube length was analyzed. Similar co-culture experiments were performed with MCF7 cells silenced with control, anti-miR26b* and anti-miR562. (D,G) Average number of tubes formed per field of view and (F,H) average tube length was analyzed. * p<0.05 ** p<0.01 vs control cells.

Next, the endothelial cell tube formation assay was performed to assess the effect of miR26b* and miR562 expression in MCF7 cells on endothelial cell tube formation (co-culture). This assay works on the premise that cells release soluble factors affecting angiogenesis into the matrigel layer containing human umbilical cord endothelial cells (HUVEC). This induces the HUVEC to form tube-like structures in the matrigel. The number of tubes formed and the average length of tubes formed were quantified and used to assess the extent of angiogenesis. Both miR26b* and miR562 over-expression in MCF7 cells resulted in an increase in average number of tubes formed (Fig. 6A,C) as well as average length of tubes formed (Fig. 6B,D). Next, a loss of function experiment was performed in which both miRs were selectively inhibited using sequence-specific inhibitors. In MCF7 cells where either miR26b* or has-miR562 was inhibited, there was a reduction in the average number of tubes formed (Fig. 6C). However, there was no difference in the average length of tubes formed (Fig. 6D). This data suggest that miR26b* and miR562 expressed in breast cancer cells can regulate tube formation by endothelial cells.

**Figure 7:**
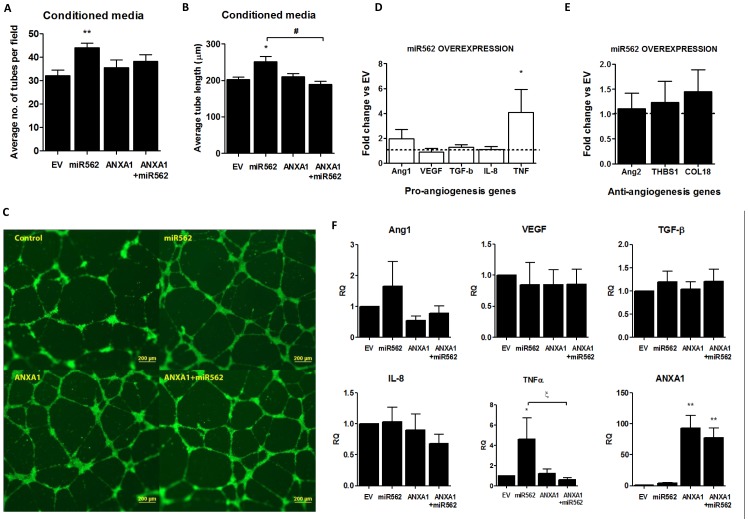
MiR562 overexpression in MCF7 cells induces secreted factors which enhance angiogenic activity of endothelial cells. (A,B) MCF7 cells were transfected with empty vector (EV), miR562, ANXA1 or cotransfected with ANXA1 and miR 562 and conditioned media was used to culture HUVEC in matrigel. Average number of tubes formed per field of view and average tube length was analyzed. (C) Representative images of the four treatments are shown. (D, E) qPCR array of pro/anti angiogenesis genes modulated after miR562 overexpression in MCF7 cells. (F) qPCR array of pro-angiogenesis genes modulated after miR562 and ANXA1 co-overexpression in MCF7 cells. * p<0.05, ** p<0.01 vs EV cells; ξ p<0.05 vs miR562 transfected cells.

To determine if miR562 could be regulating the secretion of factors from MCF7 cells which could influence endothelial cell tube formation, MCF7 conditioned media was collected from control and miR562-transfected cells and used to treat HUVEC for 24 h. Once again, treatment of HUVEC with conditioned media obtained from miR562-transfected cells significantly enhanced the number of HUVEC tubes formed as well as the length of the tubes (Fig. 7A,B), with representative images shown in Fig. 6C. Interestingly, transfection of MCF-7 cells with ANXA1 overexpression plasmid did not affect tube formation, yet cotransfection of ANXA1 with miR562 reversed the increased tube length induced with mir562 alone (Fig. 7B). To assess which possible factors could be produced by miR562-transfected MCF-7 cells, we performed real time PCR for pro-angiogenesis such as VEGF and TNFα, and anti-angiogenesis genes such as angiopoeitin-2 (ANG2) and thrombospondin (THBS1). Only TNFα was significantly increased in MCF-7 cells transfected with miR562 (Fig. 7D), with no effect on anti-angiogenesis genes (Fig. 7E). Transfection of MCF-7 cells with ANXA1 overexpression plasmid did not change any angiogenesis genes (Fig. 7F). However, transfection of ANXA1 with miR562 results in a significant inhibition in TNFα expression when compared to miR562 transfection alone, indicating that ANXA1 may inhibit the TNFα expression induced by miR562, which may regulate angiogenic activity.

In summary, we have shown that ANXA1 can modulate the expression of miR26b* and miR562, which are able to functionally down-regulate NF-κB activity both at promoter and downstream effector levels, which may lead to higher endothelial cell tube formation and lower wound healing capacity (with respect to miR562).

## Discussion

The present study provides evidence of the existence of a relationship between ANXA1, miRs and NF-κB and a unique miR signature in MCF7 breast cancer cells over-expressing ANXA1. MiR expression profiles have been previously reported in breast cancer where hsa-miR-10b, hsa-miR-125b and hsa-miR-145 were significantly downregulated in breast cancer and hsa-miR-21 and hsa-miR-155 were upregulated in breast cancer [25]. Though the microarray analysis was performed in MCF7 breast cancer cells, it might be possible to extrapolate the data of the unique miR signature obtained to other breast cancer cell lines and different breast tumor types.

More significantly, two novel miRs, hsa-miR26b* and hsa-miR562 were characterized and their targets were elucidated and experimentally validated. We focused our efforts on targets that were either NF-κB subunits or signaling molecules associated with the NF-κB pathway. Rel A encodes a subunit of the NF-κB signaling pathway that form dimers in the classical NF-κB pathway. miR26b* directly targeted the Rel A subunit at the 3′ UTR. As a result of this direct interaction, NF-κB activity decreased. This is highly significant as not many miRs have been experimentally validated to target NF-κB subunits. Our data on miR26b* is novel as no miR has been experimentally shown to target Rel A. However, p65 (protein encoded by Rel A) has been shown to induce the expression of some miRs such as hsa-miR-143 that directly targets fibronectin type III domain containing 3B (FNDC3B) that regulates adipocyte differentiation [26]. Hsa-miR-143 promoted live tumor cell invasion and metastasis, decreased cell viability and increased apoptosis of colon cancer cells upon 5′flurouracil treatment [27]. Other miRs which have been experimentally shown to target NF-κB subunits include miR146a and miR9 in inflammatory cells [28], [29]. Direct targeting of Rel A and NF-κB1 by miR26b* and miR562 respectively contributes to the understanding of how NF-κB subunits can be regulated post-transcriptionally.

It can be postulated that high expression of ANXA1 leads to a lower expression of miR26b* and miR562 which results in increased NF-κB activity. Conversely, we also show that higher expression of miR26b* and miR562 can result in reduced NF-κB activity. Other studies have reported lower NF-κB activity in MCF7 and T47D cells as compared to MDA-MB-231 cells [30], [31]. MDA-MB-231 cells are triple-negative and highly aggressive breast cancer cells that express high levels of ANXA1 [7], [32]. We have shown that endogenous levels of miR26b* and miR562 are lower in MDA-MB-231 cells where ANXA1 levels are high and NF-κB activity is constitutively active. Hence, the ANXA1-NF-κB signaling paradigm described previously by Bist et al [7] has gained clarity with miR26b* and miR562 being able to target p65 and p105 respectively.

This study presents data which can be added into the proposed model by Bist *et al* [7]. Low ANXA1 levels lead to higher levels of miR26b* which result in lower NF-κB activity in primary tumors or low-ANXA1 expressing tumors. Thus, ANXA1 can be established as playing a dualistic role in breast cancer progression. In primary tumors, non-invasive tumors or low ANXA1 expressing tumors (such as MCF7), ANXA1 may play a tumor suppressive role by keeping NF-κB activity in check via miR26b* regulation. In contrast, in metastatic or high ANXA1 expressing tumors (such as MDA-MB-231 cells), ANXA1 activates NF-κB constitutively and promotes metastasis. Once again, this provides good insight into understanding the role of ANXA1 in cancer initiation and progression.

The elucidation of molecular targets of miR26b* and miR562 has shed more light on miR regulation on NF-κB subunit expression. Our study has shown that Rel A can be regulated by a miR and this occurs post-transcriptionally. Post-translational modifications of NF-κB subunits have been studied and different groups have studied how NF-κB subunits can be modified. Post-translational modifications of NF-κB subunits have been studied by many groups but the focus has usually has been the post-translational modifications of the p65 subunit though there are some notable co-translational modifications of p105 subunit [33], [34]. It is crucial to understand that regulation of p65 by miRs occurs before the post-translational modifications of p65 and that the post-transcriptional regulation of p65 by miR26b* affects the expression and levels of p65 itself while the post-translational modifications of p65 may affect function and activity of p65.

In the past, research on NF-κB signaling has revolved around understanding the IKK complex or the factors regulating the IKK complex. There is now vast realization that NF-κB signaling is more complex and additional regulatory checkpoints exist. Not much is known about how the various NF-κB subunits are regulated and how the dimers are able to differentially affect gene transcription. This study has contributed to understanding how NF-κB subunits can be regulated post-transcriptionally and that regulation of individual NF-κB subunits may have a profound effect on NF-κB transcriptional activity.

Widespread inhibition of NF-κB activity may have detrimental effects on the cells as NF-κB plays key roles in innate immunity and various other immunological responses. The same notions apply to attempting to down-regulate expression of miR26b* or miR562 in order to down-regulate NF-κB activity in breast cancer cells.

miR26b* and miR562 have been shown to regulate endothelial cell tube formation in MCF7 cells, which relates to angiogenesis. Angiogenesis has been viewed as a promising target for cancer therapy as angiogenesis provides tumor cells with nutrients and oxygen for sustenance. Many studies have associated activation of angiogenesis to NF-κB activation and there is a paradigm that NF-κB activation can serve as an effective anti-cancer therapy. However, it may be important to reconsider this paradigm in the context of angiogenesis as it has been speculated that NF-κB activation, when performed specifically in endothelial cells, can be an effective anti-angiogenesis therapy in the treatment of cancer [35]. Studies have suggested the occurrence of the alternative signaling paradigm both *in vitro* and *in vivo* where NF-κB can inhibit angiogenesis and endothelial cell migration when activated [36], [37]. Angiostatic compounds activate NF-κB that up-regulate the expression of adhesion molecules allowing cells to escape endothelial cell anergy [38].

### Conclusions

Our studies have contributed to understanding the ANXA1-NF-κB signaling paradigm further, highlighted the regulation 2 miRNAs by ANXA1, namely miR26b* and miR562 which directly targeted an NF-κB subunit REL-A (p65) and NF-κB1 (p105), respectively. NF-κB activity was inhibited by both miR26b* and miR562, leading to the inhibition of NF-κB dependent genes which are important in wound healing/migration and angiogenesis. Functionally, higher expression of both miR26b* and miR562 in MCF7 cells lead to increased endothelial cell tube formation while inhibition of miR26b* and miR562 inhibited tube formation. Anti-miR26b* and anti-miR562 might prove to be effective angiostatic agents to inhibit tumor angiogenesis, thus curbing further growth and metastasis.

## Supporting Information

S1 Fig MiR26b* and miR562 overexpression in MCF7 cells does not modulate proliferation. MCF7 cells were transfected with empty vector (EV), miR26b* or miR562 and (A) proliferation rates analyzed using crystal violet staining daily (B) or cell cycle analysis performed using propidium iodide staining.
(TIF)
