# Supplementary figures and images for: Annexin-A1 Regulates MicroRNA-26b* and MicroRNA-562 to Directly Target NF-κB and Angiogenesis in Breast Cancer Cells
Source: PLoS One. 2014 Dec 23;9(12):e114507. doi: 10.1371/journal.pone.0114507 (PMC4275173; doi:10.1371/journal.pone.0114507)

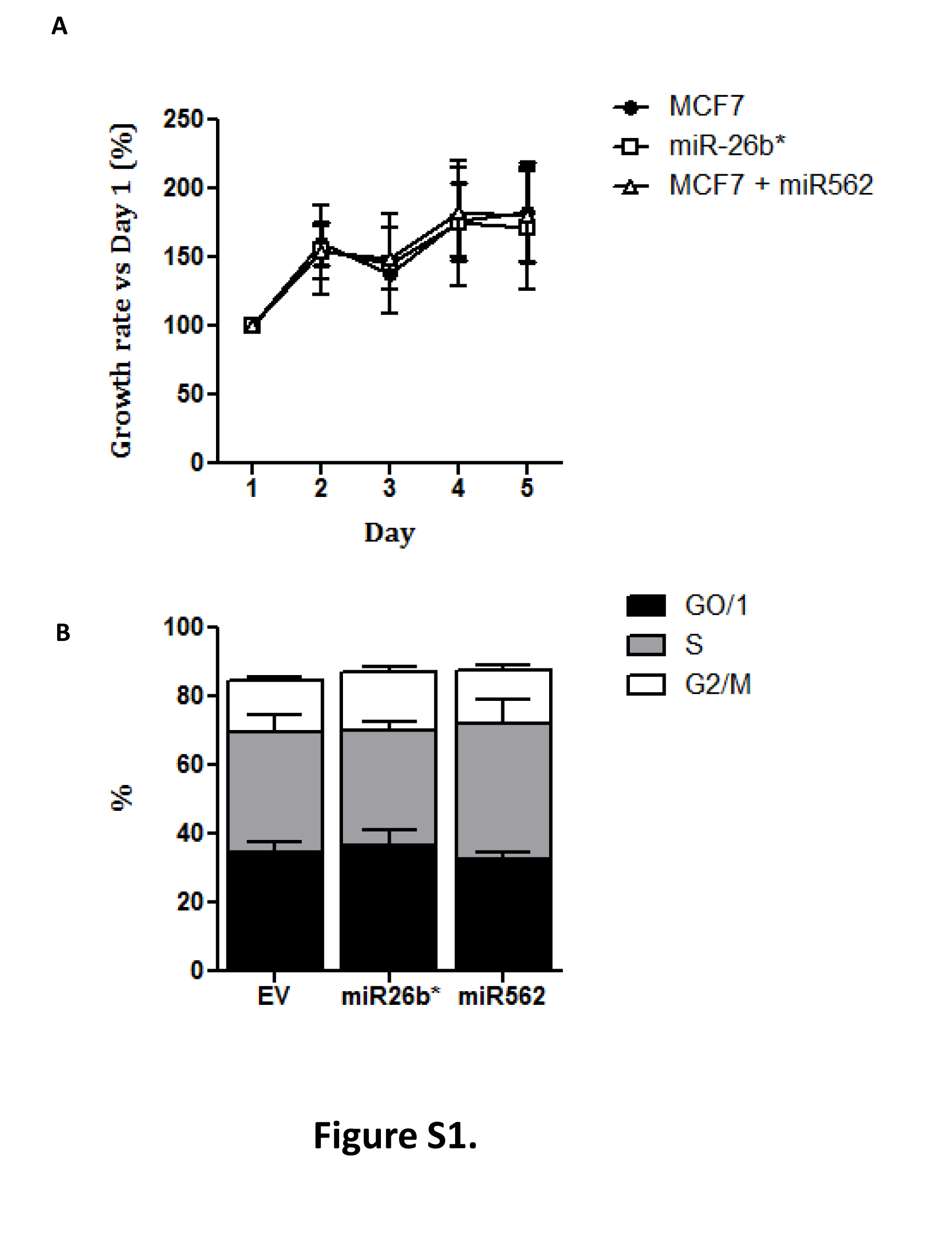

Supplement: S1 Fig — MiR26b* and miR562 overexpression in MCF7 cells does not modulate proliferation. MCF7 cells were transfected with empty vector (EV), miR26b* or miR562 and (A) proliferation rates analyzed using crystal violet staining daily (B) or cell cycle analysis performed using propidium iodide staining. (TIF) [file pone.0114507.s001.tif]
